# Predictors of households at risk for food insecurity in the United States during the COVID-19 pandemic

**DOI:** 10.1017/S1368980021000355

**Published:** 2021-01-27

**Authors:** Brianna N Lauren, Elisabeth R Silver, Adam S Faye, Alexandra M Rogers, Jennifer A Woo-Baidal, Elissa M Ozanne, Chin Hur

**Affiliations:** 1 Division of General Medicine, Department of Medicine, Columbia University Irving Medical Center, 622 W 168th St, PH9E-105, New York, NY 10032, USA; 2 Division of Digestive and Liver Diseases, Department of Medicine, Columbia University Irving Medical Center, New York, NY, USA; 3 Division of Pediatric Gastroenterology, Hepatology, and Nutrition, Department of Pediatrics, Columbia University Irving Medical Center, New York, NY, USA; 4 Department of Population Health Sciences, University of Utah, School of Medicine, Salt Lake, UT, USA; 5 Herbert Irving Comprehensive Cancer Center, Columbia University Irving Medical Center, New York, NY, USA

**Keywords:** Food insecurity, COVID-19, Mental health, Health disparities

## Abstract

**Objective:**

To examine associations between sociodemographic and mental health characteristics with household risk for food insecurity during the COVID-19 outbreak.

**Design:**

Cross-sectional online survey analysed using univariable tests and a multivariable logistic regression model.

**Setting:**

The United States during the week of 30 March 2020.

**Participants:**

A convenience sample of 1965 American adults using Amazon’s Mechanical Turk platform. Participants reporting household food insecurity prior to the pandemic were excluded from analyses.

**Results:**

One thousand two hundred and fifty participants reported household food security before the COVID-19 outbreak. Among this subset, 41 % were identified as at risk for food insecurity after COVID-19, 55 % were women and 73 % were white. On a multivariable analysis, race, income, relationship status, living situation, anxiety and depression were significantly associated with an incident risk for food insecurity. Black, Asian and Hispanic/Latino respondents, respondents with an annual income <$100 000 and those living with children or others were significantly more likely to be newly at risk for food insecurity. Individuals at risk for food insecurity were 2·60 (95 % CI 1·91, 3·55) times more likely to screen positively for anxiety and 1·71 (95 % CI 1·21, 2·42) times more likely to screen positively for depression.

**Conclusions:**

An increased risk for food insecurity during the COVID-19 pandemic is common, and certain populations are particularly vulnerable. There are strong associations between being at risk for food insecurity and anxiety/depression. Interventions to increase access to healthful foods, especially among minority and low-income individuals, and ease the socioemotional effects of the outbreak are crucial to relieving the economic stress of this pandemic.

As the economic consequences of the COVID-19 pandemic ripple through the United States, foodbanks across the country have reported unprecedented demand, with many distribution centres falling short of community need^(1,2)^. Unemployment has skyrocketed to Depression-era rates^(3)^, and schools offering free or reduced-price meals have closed, potentially leaving households more vulnerable to food insecurity than ever before.

Emerging national data demonstrate an increase in household food insecurity across the country. During the week of 7 May 2020, the US Census Bureau received almost 42 000 responses to their Household Pulse Survey. This survey estimated that 10 % of US adults experienced food scarcity in the past 7 d^(4)^, although other national surveys have reported even higher numbers. The COVID Impact Survey, conducted by NORC at the University of Chicago for the Data Foundation, recruited a nationally representative sample of 2190 adults during the week of 20 April. Within this sample, 28 % reported often or sometimes worrying about food running out within the past 30 d. In addition, 22 % reported that food often or sometimes did not last and they did not have money to buy more^(5)^. Finally, about 9000 non-elderly adults responded to the Urban Institute’s nationally representative Health Reform Monitoring Survey between 25 March and 10 April, which found that 21·9 % of respondents reported household food insecurity^(6)^.

Past work examining food insecurity in the wake of major natural disasters in the United States has found that risk factors for becoming food-insecure include race/ethnicity (particularly black and Hispanic/Latino individuals), lower household income, poorer mental health and poorer physical health^(7,8)^. The COVID-19 pandemic has disproportionately impacted these same communities^(9)^. The Health Reform Monitoring Survey found that black and Hispanic adults were more than twice as likely to report household food insecurity compared to non-Hispanic white adults (33·9 % and 33·3 % *v*. 16·3 %, respectively)^(6)^. During the week of 19 March 2020, researchers from the University of Michigan School of Public Health found that 44 % of low-income US adults with <250 % of the Federal Poverty Line were food-insecure, and another 20 % reported only marginal food security^(10)^. As with past major disasters, the pandemic exacerbates existing systemic inequalities in access to material resources that support health and well-being.

Food insecurity is associated with poor health outcomes, including CVD, BMI >30 and diabetes^(11,12)^. These conditions are associated with developing severe COVID-19 symptoms^(13)^. Thus, identifying those who are most vulnerable to household food insecurity amid the economic fallout of COVID-19 is of crucial public health importance. To this end, we conducted a cross-sectional survey of American adults to assess sociodemographic characteristics associated with incident household food insecurity in the wake of the COVID-19 outbreak.

## Methods

We conducted a cross-sectional survey of American adults through Amazon’s Mechanical Turk (MTurk), an online labour market of over 225 000 US workers who complete online tasks and surveys^(14)^. Participants were recruited using convenience sampling. MTurk workers were invited to take part in an online survey, administered using Qualtrics, that included questions regarding demographics, social distancing, food security before and after the COVID-19 outbreak and anxiety and depression (described below in Measures and in the Supplementary Material). Surveys were completed between 30 March 2020 and 2 April 2020, after many states had imposed stay-at-home orders^(15)^. Participants were compensated with $0·50 for completing the survey, which took 10–15 min to complete.

### Measures

#### Demographics

Demographic variables included age, gender, race/ethnicity, annual household income, postal code, relationship status, employment status at time of survey and whether participants lived with children of age <18.

#### Household risk for food insecurity

We assessed household risk for food insecurity using a validated two-item screen^(16)^. Households at risk for food insecurity were defined as those with responses of ‘sometimes true’ or ‘often true’ for either or both items. Participants reported answers to each of these questions for the periods before and after the COVID-19 outbreak.

#### Anxiety and depression

Anxious and depressive symptoms were assessed as part of the PROMIS-29 +2 (PROPr) scale^(17)^. Participant responses were expected to reflect their mental state at the time of the survey. Based on past work^(18)^, anxiety subscale *t*-scores ≥62·3 were categorized as positive for anxiety, and depression subscale *t*-scores ≥59·9 were categorized as positive for depression.

#### Additional measures

Additional survey questions asked respondents about the effect of COVID-19 on diet, exercise and health-related quality of life. A descriptive report of findings from these measures is currently under review. These variables were not included in the present analyses as they were unrelated to our primary aims.

### Analysis

#### Univariable analysis

The study population was restricted to respondents with household food security before the COVID-19 outbreak. We compared baseline demographic characteristics and anxiety and depression across respondents with and without incident household risk for food insecurity. We used *χ* ^2^ tests of independence for categorical variables, and Fisher’s exact test when expected values were ≤5. Respondents with missing data for a particular analysis were excluded. Statistical significance was defined as *P* < 0·05.

#### Multivariable analysis

We constructed a multivariable logistic regression model assessing household risk for food insecurity as our outcome variable. Gender and age were included as covariables a priori. We included additional demographic characteristics if *P* < 0·20 on univariable analyses. Respondents with missing data for included variables were excluded from the model. Statistical testing was performed in R (version 3.6.3).

## Results

A total of 1965 participants across the United States completed our survey between 30 March and 2 April 2020. Among the total survey respondents, 1250 (63 %) reported household food security before the outbreak. Subsequent analyses were based on this subset of the population.

**Fig. 1:**
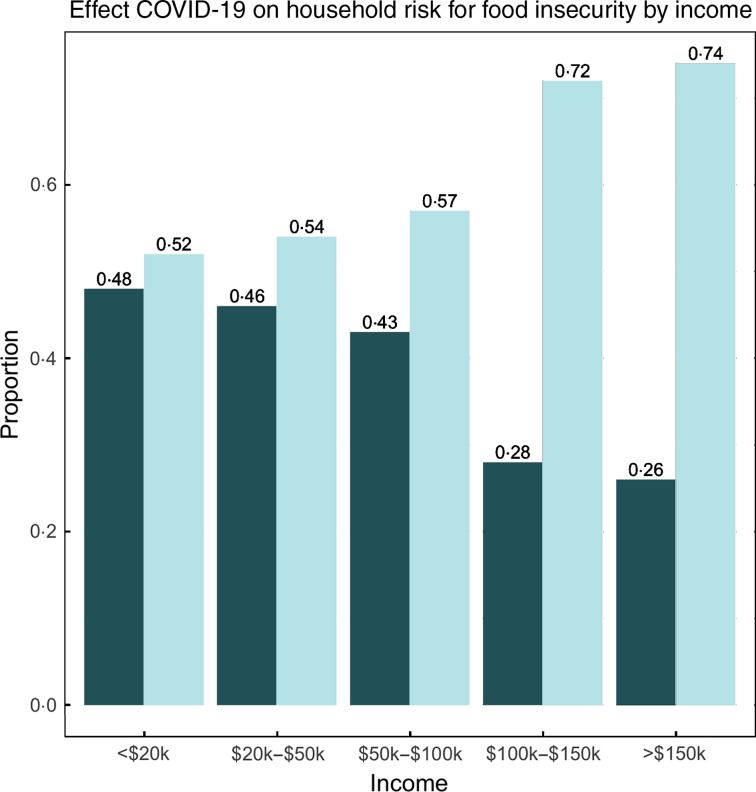
Effect of COVID-19 on household risk for food insecurity by income (*n* 1250). 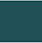, became at risk for food insecurity; 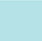, stayed food-secure

Within the population of food-secure participants pre-COVID-19, 55 % identified as female, 72 % were between the ages of 25 and 55, 73 % were white, 11 % were Asian, 6 % were black and 6 % were Hispanic. In addition, 65 % reported an annual household income of >$50 000, and 63 % reported full-time employment at the time of the survey. Roughly 41 % of previously food-secure participants were identified as at risk for food insecurity after the outbreak (Table 1). Figure 1 illustrates the change in household risk for food insecurity by annual income level after the COVID-19 outbreak. Respondents at each income level reported incident risk for food insecurity, ranging from 26 % to 48 % of the population at different income levels.

**Table 1:** Summary of sample characteristics

	Count	%
Total	*n* 1250	
Household at risk for food insecurity after outbreak		
No	737	59·0
Yes	513	41·0
Gender		
Man	527	42·2
Woman	690	55·2
Other	7	0·6
Missing	26	2·1
Age		
18–24	127	10·2
25–34	384	30·7
35–44	317	25·4
45–54	202	16·2
55–64	137	11·0
65+	82	6·6
Missing	1	0·1
Race		
White	918	73·4
Black	73	5·8
Asian	142	11·4
Hispanic	76	6·1
Other, including multiracial	37	3·0
Missing	4	0·3
Annual income		
<$20 k	87	7·0
$20–$50 k	354	28·3
$50–$100 k	521	41·7
$100–$150 k	195	15·6
>$150 k	91	7·3
Missing	2	0·2
Employment status		
Full-time	784	62·7
Part-time	189	15·1
Student	59	4·7
Unemployed	210	16·8
Missing	8	0·6
Relationship status		
Single	308	24·6
Divorced/separated/widowed	117	9·4
Committed relationship	119	9·5
Married/co-habitating	702	56·2
Missing	4	0·3
Household composition		
Alone	237	19·0
With children	375	30·0
With others	635	50·8
Missing	3	0·2
State’s stay-at-home orders		
No	289	23·1
Yes	947	75·8
Missing	14	1·1
Region		
Northeast	245	19·6
West	307	24·6
South	435	34·8
Midwest	249	19·9
Missing	14	1·1
Anxiety screen		
Negative	829	66·3
Positive	419	33·5
Missing	2	0·2
Depression screen		
Negative	964	77·1
Positive	280	22·4
Missing	6	0·5

On an univariable analysis, age, race, income, living situation, anxiety and depression were most strongly associated with incident household risk for food insecurity (Table 2). Respondents aged 25–34 represented a larger percentage of the population with incident household risk for food insecurity compared to the population without (33 % *v*. 29 %, respectively; *P* < 0·01). Racial/ethnic minorities and respondents with lower incomes also represented larger proportions of individuals with incident household risk for food insecurity (*P* < 0·01). Respondents living with children or others represented the largest proportions of the population with an incident risk for food insecurity (35 % and 51 %, respectively; *P* < 0·01). Among the participants identified as at risk for food insecurity, 49 % screened positive for anxiety compared to 23 % of participants who remained food-secure (*P* < 0·01). Similarly, 33 % of participants identified as at risk for food insecurity screened positive for depression compared to 15 % of participants who remained food-secure (*P* < 0·01). Employment status and region were also significantly associated with incident household risk for food insecurity (*P* = 0·049 and 0·015, respectively).

**Table 2:** Summary of univariable analysis of sample characteristics by household risk of food insecurity

Characteristics	Became at risk for food insecurity*		Remained food-secure*		*P*-value†
	*n*	%	*n*	%	
	513		737		
Age, years					
18–24	53	10·3	74	10·1	0·001
25–34	170	33·1	214	29·1	
35–44	136	26·5	181	24·6	
45–54	91	17·7	111	15·1	
55–64	45	8·8	92	12·5	
65+	18	3·5	64	8·7	
Gender					
Man	212	42·2	315	43·6	0·894
Woman	287	57·2	403	55·8	
Other	3	0·6	4	0·6	
Race/ethnicity					
White	334	65·2	584	79·6	<0·001
Black	43	8·4	30	4·1	
Asian	78	15·2	64	8·7	
Hispanic	40	7·8	36	4·9	
Other, including multiracial	17	3·3	20	2·7	
Household income					
<$20 k	42	8·2	45	6·1	<0·001
$20–$50 k	164	32·1	190	25·8	
$50–$100 k	226	44·2	295	40·0	
$100–$150 k	55	10·8	140	19·0	
>$150 k	24	4·7	67	9·1	
Region					
Northeast	93	18·4	152	20·8	0·015
West	135	26·7	172	23·5	
South	194	38·4	241	33·0	
Midwest	83	16·4	166	22·7	
Relationship status					
Single	113	22·0	195	26·6	0·107
Divorced/separated/widowed	42	8·2	75	10·2	
Committed relationship	54	10·5	65	8·9	
Married/co-habitating	304	59·3	398	54·3	
Employment status					
Full-time	327	64·0	457	62·5	0·049
Part-time	90	17·6	99	13·5	
Student	19	3·7	40	5·5	
Unemployed	75	14·7	135	18·5	
Impact of COVID-19 on employment					
Became unemployed	35	6·8	51	7·0	1·00
No change in employment	476	93·2	680	93·0	
State’s stay-at-home orders					
Enacted	387	76·6	560	76·6	1·00
Not enacted	118	23·4	171	23·4	
Living situation					
Alone	75	14·7	162	22·0	0·001
With children	177	34·6	198	26·9	
With others	259	50·7	376	51·1	
Anxiety screen					
Positive	248	48·5	171	23·2	<0·001
Negative	263	51·5	566	76·8	
Depression screen					
Positive	168	33·0	112	15·2	<0·001
Negative	341	67·0	623	84·8	

* Percentages may not sum to 100 due to missing cases.

† Two-tailed *P*-values.

**Fig. 2:**
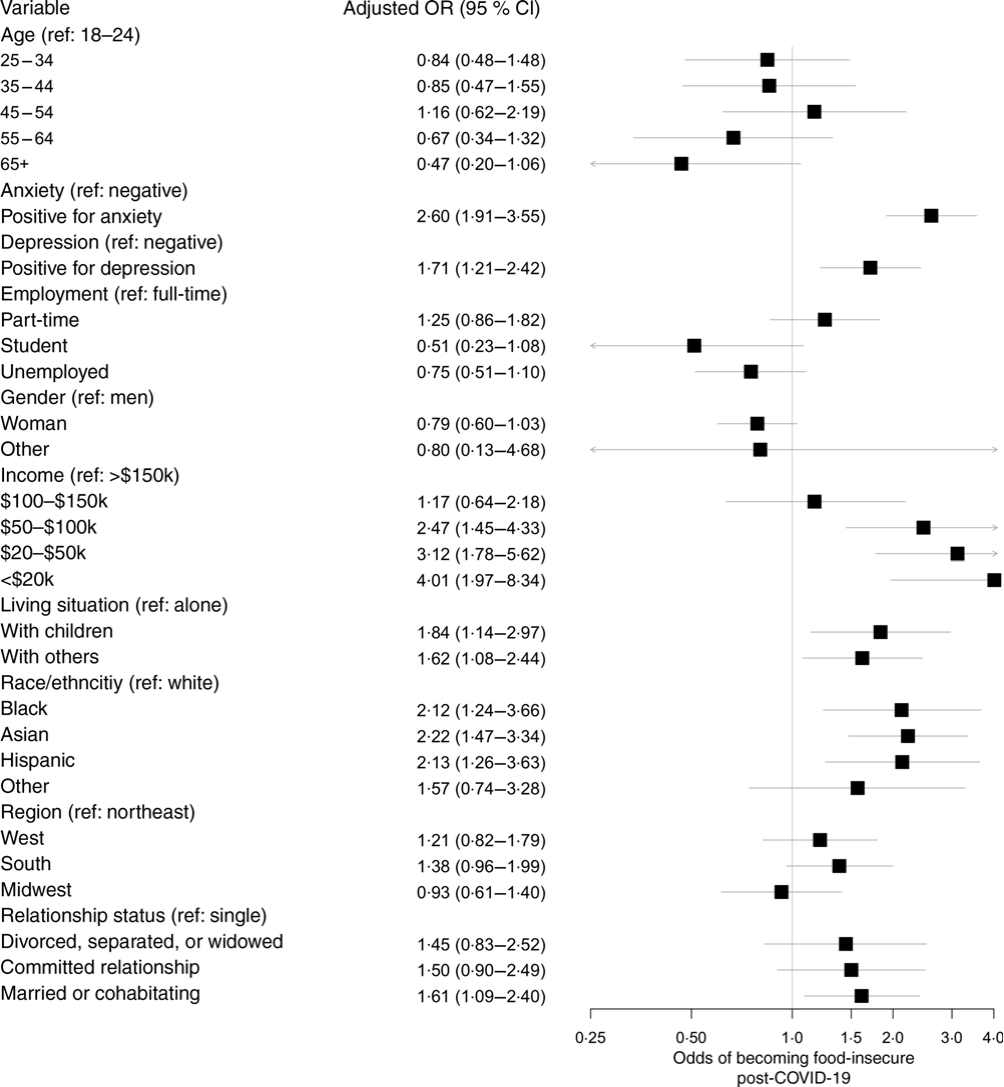
Summary of multivariable analysis of sample characteristics by household risk for food insecurity (*n* 1250)

On a multivariable analysis, race, income, living situation, relationship status, anxiety and depression were all independently associated with an incident risk for food insecurity (Fig. 2). Black, Asian and Hispanic respondents were significantly more likely to experience an incident risk for food insecurity compared to white respondents (adj. OR (95 % CI): 2·12 (1·24, 3·66); 2·22 (1·47, 3·34); and 2·13 (1·26, 3·63), respectively). Relative to those with an annual income >$150 000, those with annual incomes <$100 000 were significantly more likely to be newly at risk for food insecurity, with the greatest effect observed for those with annual incomes <$20 000 (adj. OR 4·01, 95 % CI 1·97, 8·34). In addition, respondents who were married or co-habitating were significantly more likely to experience an incident household risk for food insecurity compared to single/casually dating respondents (OR 1·61, 95 % CI 1·09, 2·40). Respondents living with children or others were also significantly more likely to be newly at risk for food insecurity compared to those living alone (adj. OR (95 % CI): 1·84 (1·14, 2·97) and 1·62 (1·08, 2·44), respectively). Finally, respondents screening positive for anxiety or depression were significantly more likely to report an incident household risk for food insecurity (OR (95 % CI): 2·60 (1·91, 3·55) and 1·71 (1·21, 2·42), respectively).

## Discussion

In this cross-sectional survey of American adults, we found that 41 % of previously food-secure respondents became newly at risk for food insecurity following the COVID-19 outbreak. By contrast, the USDA reported that only 11 % of total US households were food-insecure in 2018^(19)^. Our results suggest that this percentage will rise steeply as many more households become newly vulnerable during COVID-19. The risk for incident food insecurity was significantly higher among black, Asian and Hispanic participants, as well as respondents who were married or co-habitating, living with children or others or reported household incomes <$100 000 at the time of the survey. In addition, there was a significant association between incident household risk for food insecurity and screening positive for anxiety or depression. These results suggest that the COVID-19 pandemic exacerbates existing societal inequalities, with potential downstream consequences for health and pandemic recovery efforts.

Emerging data suggest that black and Hispanic individuals are disproportionately impacted by the COVID-19 pandemic^(20)^. In New York City, the epicentre of COVID-19 in the United States at the time of this study, black and Hispanic/Latino COVID-19 patients are twice as likely to die of the disease than whites^(9)^. Moreover, black and Hispanic populations are disproportionately affected by CVD^(21)^, which is associated both with food insecurity^(11,12)^ and severe COVID-19 disease^(13)^. While the results of our study provide further evidence of these disparities, it also suggests vulnerability among Asian populations as well. Black, Hispanic and Asian individuals were over twice as likely to be newly at risk for food insecurity, even after adjusting for other factors such as income and employment status. Targeted efforts to increase household food security among black, Hispanic and Asian communities are needed to alleviate the disproportionate impact of COVID-19 on people of colour.

In addition to highlighting existing disparities, our results underscore the far-reaching impacts of COVID-19. Among respondents with an annual household income between $50 000 and $100 000, 43 % experienced an incident household risk for food insecurity after the outbreak. This proportion was lower for respondents with an annual household income >$150 000, but still remained at a surprising 26 %. By contrast, the USDA reported only 5·4 % of households with annual incomes at or above 185 % of the federal poverty line ($47 110 for a family of four) experienced household food insecurity in 2018^(19)^. While the rate of risk for food insecurity we found (41 %) was lower than those reported a week earlier by Wolfson and Leung^(10)^, our sample included both high- and low-income individuals and excluded those who had been food-insecure before the COVID-19 outbreak. As a result, we were able to distinguish incident from chronic food insecurity, highlighting the extent and acute nature of the pandemic’s effects – many Americans, even those in higher income brackets, are becoming newly vulnerable to household food insecurity.

Our finding regarding the relationship between risk for food insecurity and anxiety/depression is consistent with past work documenting a bidirectional relationship between food insecurity and poor mental health and emotional well-being^(22)^. More specifically, our study found that respondents experiencing an incident household risk for food insecurity were roughly twice as likely to experience anxiety or depression, even after controlling for other factors. This is of particular concern as previous studies have demonstrated the negative effect both food insecurity and parental depressive symptoms have on the physical health and future developmental, behavioural and psychiatric function of children^(23,24)^. Given that the relationship between poor mental health and food insecurity is exacerbated by social isolation^(25)^, increasing the accessibility of mental healthcare during this time may augment more direct efforts to bolster food security and, in turn, support child health and development initiatives as well.

Our study has limitations. First, our cross-sectional approach precludes causality inferences and relies on retrospective reports of food insecurity prior to the COVID-19 outbreak. Second, we collected data through MTurk, potentially creating a response bias and limiting the generalizability of our findings. Our sample included proportionally fewer black and Hispanic individuals (6 % *v*. 13 % and 6 % *v*. 18 %, respectively) and proportionally more Asian individuals (11 % *v*. 6 %) than the larger US population^(26)^. The survey did not specify whether participants were immigrants or born in the United States, limiting our ability to analyse the risk for food insecurity among immigrants, a particularly vulnerable subgroup^(27)^. Our data collection method also restricted our sample to English-competent respondents with internet access. Those segments of the population with limited English proficiency^(28)^ and those who are least likely to have internet access (people of colour, low-income adults, those without high school diplomas and those living in rural areas)^(29)^ were, therefore, invisible to our study. Moreover, our data were collected in late March, when the economic impacts of the outbreak were just beginning to affect many nationwide. We hypothesize that this may partially explain the unexpected result that unemployment was not a predictor of an incident risk for food insecurity. Although the validated food insecurity screening questions that we used in our study specifically referenced an ability to afford food (rather than an inability to buy or find food due to shortages), our finding of an increased risk of food insecurity among those with an annual income between $50 000 and $100 000 is also surprising. Given the nationwide food shortages that were occurring at this time, it is possible that participants’ inability to find food could have led to an overestimation of their risk for food insecurity. Additionally, because we did not specify a timeframe for these earnings, participants’ responses may reflect historical income rather than concurrent changes in annual income resulting from the pandemic. The documented stress and anxiety also could have influenced the way participants responded to the screening questions. Lastly, the use of the two-item screener rather than the eighteen-item US Household Food Security Module limited our ability to definitively classify participants’ household food security. Nevertheless, the screener allowed us to quickly identify those at risk for food insecurity and glimpse into the acute changes happening in US households. Continued research on the effects of the pandemic on food insecurity and how best to mitigate many households’ newfound risk, particularly in larger community-based samples, is necessary to fully understand its impacts and respond effectively.

Our study quantifies the effects of COVID-19 on the risk for food insecurity among a subset of US households and highlights associated risk factors. Our results suggest that the economic and socioemotional fallout of the pandemic has made many households newly vulnerable, with minorities, low-income individuals and those with poorer mental health at higher risk. As such, targeted relief efforts are needed as we look towards economic recovery.

## Supplementary material

For supplementary material accompanying this paper visit http://dx.doi.org/10.1017/S1368980021000355. click here to view supplementary material
