# Supplementary material for: Predictors of households at risk for food insecurity in the United States during the COVID-19 pandemic
Source: Public Health Nutr. 2021 Jan 27:1–8. doi: 10.1017/S1368980021000355 (PMC8207551; doi:10.1017/S1368980021000355)
Supplement: Supplementary file 1 [file S1368980021000355sup001.docx]

**Predictors of households at risk for food insecurity in the United States during the**

**COVID-19 pandemic**

**Supplement:** Transcript of survey administered to participants.

**eMethods: Survey Transcript**

**Eligibility**

Are you at least 18 years old?

- Yes (1)
- No (0)

Are you comfortable reading and writing in English?

- Yes (1)
- No (0)

**Demographics**

What is your age?

- 18 - 24 (1)
- 25 - 34 (2)
- 35 - 44 (3)
- 45 - 54 (4)
- 55 - 64 (5)
- 65 - 74 (6)
- 75 - 84 (7)
- 85 or older (8)

What is your gender?

- Man (1)
- Woman (2)
- Non-binary (3)
- My gender is not listed above: (4) ________________________________________________

What is your race/ethnicity?

- Black or African-American (1)
- Asian or Asian-American (2)
- Hispanic or Latino (3)
- American Indian or Alaska Native (4)
- Native Hawaiian or Other Pacific Islander (5)
- White (6)
- Biracial or multiracial (7)
- My race is not listed above: (8) ________________________________________________

What is your relationship status?

- Married (1)
- Living or cohabitating with partner (2)
- Widowed (3)
- Divorced (4)
- Separated (5)
- Casually dating (7)
- Currently single (8)
- In committed relationship (9)

What is your employment status?

- Employed full-time (1)
- Employed part-time (2)
- Student (3)
- Unemployed (4)

If employed or student:

|  | Before COVID-19 | After COVID-19 |
| --- | --- | --- |
|  |  |  |
| On average, how many days per week did you/do you work or learn from home? (1) | - Always - Most of the time - About half the time - Sometimes - Never | - Always - Most of the time - About half the time - Sometimes - Never |

If unemployed:

Were you unemployed before the COVID-19 outbreak?

- Yes (1)
- No (2)

Does anyone live with you? Please select all that apply.

- No, I live alone. (1)
- Children (under the age of 18) (2)
- Spouse or partner (3)
- Older adult relatives (4)
- Roommate (5)
- Other: (6) ________________________________________________

If children live with you:

Are your children currently attending school **in person**?

- Yes (1)
- No (2)

If children are **not** currently attending school in person:

Are your children continuing their learning from home?

- Yes (1)
- No (2)

If children are **not** currently attending school in person:
Did your children stop attending school **in person** due to the novel coronavirus (COVID-19) outbreak?

- No, they learned from home before the outbreak (1)
- Yes, I removed them from school (2)
- Yes, the school switched to remote learning (3)

Which of the following best describes your annual household income?

- Less than $10,000 (1)
- $10,000 - $19,999 (2)
- $20,000 - $29,999 (3)
- $30,000 - $39,999 (4)
- $40,000 - $49,999 (5)
- $50,000 - $59,999 (6)
- $60,000 - $69,999 (7)
- $70,000 - $79,999 (8)
- $80,000 - $89,999 (9)
- $90,000 - $99,999 (10)
- $100,000 - $149,999 (11)
- More than $150,000 (12)

What is your postal code, or the postal code of the nearest major city to you?

What kinds of social distancing measures are **people in your community** practicing because of the novel coronavirus (COVID-19)? Please select all that apply.

- School and university buildings are closed (1)
- Restaurants, bars, and cafes are closed or take-out and delivery only (2)
- Concert venues, movie theaters, gyms, sports arenas, etc. are closed (3)
- Shelter in place (4)
- Movement is limited to essential functions only (health-related, work-related, nutrition-related movement is allowed) (5)
- Companies are encouraging working from home (6)
- Public transit disruptions (e.g., less frequent bus or subway service) (7)
- Other measures not listed above: (8) ________________________________________________

What kinds of social distancing measures are **you** practicing because of the novel coronavirus (COVID-19)? Please select all that apply.

- Not sending my children to school (1)
- Avoiding bars, restaurants, and cafes (2)
- Avoiding concert venues, movie theaters, sports arenas, etc. (3)
- Leaving my home only for essential functions (5)
- Working or learning from home (6)
- I am not taking public transit (e.g., bus, subway) (7)
- Other measures not listed above: (8) ________________________________________________

**Diet**

The following questions ask about your **weekly** habits:

|  | Before COVID-19 | After COVID-19 |
| --- | --- | --- |
|  |  |  |
| How many times per week did/do you exercise? | - 0 - 1-2 - 3-4 - 5-6 - 7+ | - 0 - 1-2 - 3-4 - 5-6 - 7+ |
| How many times per week did/do you eat lunch prepared away from home? | - 0 - 1-2 - 3-4 - 5-6 - 7+ | - 0 - 1-2 - 3-4 - 5-6 - 7+ |
| How many times per week did/do you eat dinner prepared away from home? | - 0 - 1-2 - 3-4 - 5-6 - 7+ | - 0 - 1-2 - 3-4 - 5-6 - 7+ |
| How many times per week did/do you order take-out or delivery? | - 0 - 1-2 - 3-4 - 5-6 - 7+ | - 0 - 1-2 - 3-4 - 5-6 - 7+ |
| How many times per week did/do you eat meals from a fast food or pizza place? | - 0 - 1-2 - 3-4 - 5-6 - 7+ | - 0 - 1-2 - 3-4 - 5-6 - 7+ |
| How many times per week did/do you eat frozen meals or frozen pizza? | - 0 - 1-2 - 3-4 - 5-6 - 7+ | - 0 - 1-2 - 3-4 - 5-6 - 7+ |
| How many times per week did/do you drink regular (not diet) soft drinks or soda? | - 0 - 1-2 - 3-4 - 5-6 - 7+ | - 0 - 1-2 - 3-4 - 5-6 - 7+ |
| How many times per week did/do you drink other sugar-sweetened beverages such as fruit-flavored drinks, sports drinks, or energy drinks? | - 0 - 1-2 - 3-4 - 5-6 - 7+ | - 0 - 1-2 - 3-4 - 5-6 - 7+ |
| How many times per week did/do you drink alcohol? | - 0 - 1-2 - 3-4 - 5-6 - 7+ | - 0 - 1-2 - 3-4 - 5-6 - 7+ |

**Before** the coronavirus outbreak, where did you exercise? Please select all that apply.

- Inside my home (1)
- Community gym (e.g., gym in apartment building or living community) (2)
- Commercial gym (e.g., YMCA, Planet Fitness, yoga or dance studio) (3)
- Outside (e.g., park, trails, sidewalks) (4)
- Another location not listed above: (5) ________________________________________________
- Not applicable, I didn't exercise regularly (6)

**After** the coronavirus outbreak, where do you exercise? Please select all that apply.

- Inside my home (1)
- Community gym (e.g., gym in apartment building or living community) (2)
- Commercial gym (e.g., YMCA, Planet Fitness, yoga or dance studio) (3)
- Outside (e.g., park, trails, sidewalks) (5)
- Another location not listed above: (6) ________________________________________________
- Not applicable, I didn't exercise regularly (7)

The following questions ask about your **daily** habits:

|  | Before COVID-19 | After COVID-19 |
| --- | --- | --- |
|  |  |  |
| How many full meals did/do you eat? (daily_meals) | - 0 - 1 - 2 - 3 - 4+ | - 0 - 1 - 2 - 3 - 4+ |
| How many snacks did/do you eat between meals? (daily_snacks) | - 0 - 1 - 2 - 3 - 4+ | - 0 - 1 - 2 - 3 - 4+ |
| How many times did/do you eat vegetables per day? Do not include French fries, fried potatoes, or potato chips. (daily_veg) | - 0 - 1 - 2 - 3 - 4+ | - 0 - 1 - 2 - 3 - 4+ |
| How many times did/do you eat fruit exper day? Include fresh, frozen, canned, or 100% fruit juice. (daily_fruit) | - 0 - 1 - 2 - 3 - 4+ | - 0 - 1 - 2 - 3 - 4+ |
| How many times did/do you eat meat per day? (daily_meat) | - 0 - 1 - 2 - 3 - 4+ | - 0 - 1 - 2 - 3 - 4+ |

The following questions ask about your ability to buy food:

|  | Before COVID-19 | | | After COVID-19 | | |
| --- | --- | --- | --- | --- | --- | --- |
|  | Often true (1) | Sometimes true (2) | Never true (3) | Often true (1) | Sometimes true (2) | Never true (3) |
| You worried that your food would run out before you got money to buy more. |  |  |  |  |  |  |
| The food you bought just didn't last and you didn't have money to get more. |  |  |  |  |  |  |

diet_other Are there any other ways your diet and/or exercise has changed since the coronavirus outbreak that you did not get a chance to share in the previous questions?

________________________________________________________________

________________________________________________________________

________________________________________________________________

________________________________________________________________

________________________________________________________________

**Quality of Life (PROPr)**

Please indicate your level of physical ability regardless of whether there are outside restrictions (e.g., shelter in place or stay at home orders) on your movement:

|  | Without any difficulty (1) | With a little difficulty (2) | With some difficulty (3) | With much difficulty (4) | Unable to do (5) |
| --- | --- | --- | --- | --- | --- |
| Are you able to do chores such as vacuuming or yard work? (1) |  |  |  |  |  |
| Are you able to go up and down stairs at a normal pace? (2) |  |  |  |  |  |
| Are you able to go for a walk of at least 15 minutes? (3) |  |  |  |  |  |
| Are you able to run errands and shop? (4) |  |  |  |  |  |

In the past 7 days...

|  | Never (1) | Rarely (2) | Sometimes (3) | Often (4) | Always (5) |
| --- | --- | --- | --- | --- | --- |
| I felt fearful (1) |  |  |  |  |  |
| I found it hard to focus on anything other than my anxiety (2) |  |  |  |  |  |
| My worries overwhelmed me (4) |  |  |  |  |  |
| I felt uneasy (5) |  |  |  |  |  |

In the past 7 days...

|  | Never (1) | Rarely (2) | Sometimes (3) | Often (4) | Always (5) |
| --- | --- | --- | --- | --- | --- |
| I felt worthless (1) |  |  |  |  |  |
| I felt helpless (2) |  |  |  |  |  |
| I felt depressed (4) |  |  |  |  |  |
| I felt hopeless (5) |  |  |  |  |  |

During the past 7 days...

|  | Not at all (1) | A little bit (2) | Somewhat (3) | Quite a bit (4) | Very much (5) |
| --- | --- | --- | --- | --- | --- |
| I feel fatigued (1) |  |  |  |  |  |
| I have trouble starting things because I am tired (2) |  |  |  |  |  |

In the past 7 days...

|  | Not at all (1) | A little bit (2) | Somewhat (3) | Quite a bit (4) | Very much (5) |
| --- | --- | --- | --- | --- | --- |
| How run-down did you feel on average? (1) |  |  |  |  |  |
| How fatigued did you feel on average? (2) |  |  |  |  |  |

In the past 7 days my sleep quality was

- Very poor (1)
- Poor (2)
- Fair (3)
- Good (4)
- Very good (5)

In the past 7 days...

|  | Not at all (1) | A little bit (2) | Somewhat (3) | Quite a bit (4) | Very much (5) |
| --- | --- | --- | --- | --- | --- |
| My sleep was refreshing (1) |  |  |  |  |  |
| I had a problem with my sleep (2) |  |  |  |  |  |
| I had difficulty falling asleep (3) |  |  |  |  |  |

|  | Never (1) | Rarely (2) | Sometimes (3) | Usually (4) | Often (5) |
| --- | --- | --- | --- | --- | --- |
| I have trouble doing all of my regular leisure activities with others (1) |  |  |  |  |  |
| I have trouble doing all of the family activities that I want to do (2) |  |  |  |  |  |
| I have trouble doing all of my usual work (include work at home) (3) |  |  |  |  |  |
| I have trouble doing all of the activities with friends that I want to do (4) |  |  |  |  |  |

In the past 7 days...

|  | Not at all (1) | A little bit (2) | Somewhat (3) | Quite a bit (4) | Very much (5) |
| --- | --- | --- | --- | --- | --- |
| How much did pain interfere with your day to day activities? (1) |  |  |  |  |  |
| How much did pain interfere with your work around the home? (2) |  |  |  |  |  |
| How much did pain interfere with your ability to participate in social activities? (3) |  |  |  |  |  |
| How much did pain interfere with your household chores? (4) |  |  |  |  |  |

In the past 7 days...

|  | Not at all (1) | A little bit (2) | Somewhat (3) | Quite a bit (4) | Very much (5) |
| --- | --- | --- | --- | --- | --- |
| I have been able to concentrate (1) |  |  |  |  |  |
| I have been able to remember to do things, like take medicine or buy something I needed (2) |  |  |  |  |  |

In the past 7 days...

|  | 0  **No Pain** (1) | 1 (2) | 2 (3) | 3 (4) | 4 (5) | 5 (6) | 6 (7) | 7 (8) | 8 (9) | 9 (10) | 10  **Worst pain imaginable** (11) |
| --- | --- | --- | --- | --- | --- | --- | --- | --- | --- | --- | --- |
| How would you rate your pain on average? (1) |  |  |  |  |  |  |  |  |  |  |  |

Do you have any comments about your experience taking this survey?

________________________________________________________________

________________________________________________________________

________________________________________________________________

________________________________________________________________

________________________________________________________________
